# Disseminated Superficial Actinic Porokeratosis (DSAP): A Case Report Highlighting the Clinical, Dermatoscopic, and Pathology Features of the Condition

**DOI:** 10.7759/cureus.26923

**Published:** 2022-07-16

**Authors:** Muhammad Umer Waqar, Philip R Cohen, Simona Fratila

**Affiliations:** 1 Dermatology, University of Oradea, Oradea, ROU; 2 Dermatology, University of California, Davis Medical Center, Sacramento, USA

**Keywords:** disseminated superficial actinic porokeratosis (dsap), porokeratosis, mibelli, linear, genitogluteal, dermatoscopy, cornoid lamella

## Abstract

Porokeratosis describes a heterogenic group of keratinization disorders in which lesions are papules and plaques that demonstrate central atrophy surrounded by a hyperkeratotic margin. Clinical variants include not only porokeratosis of Mibelli, but also disseminated superficial, disseminated actinic superficial, linear, punctate, and palmaris et plantaris disseminata. Porokeratosis has a risk of malignant transformation. A woman with disseminated superficial actinic porokeratosis (DSAP) whose lesions presented as pruritic plaques and papules is described. The diagnosis was suspected clinically, supported by dermoscopy findings, and confirmed histologically. The condition-associated pruritus was managed symptomatically; her skin lesions will be monitored clinically. Clinical manifestations, dermatoscopic features, pathology findings, and treatment options for DSAP are summarized.

## Introduction

Porokeratosis refers to a group of keratinization disorders that are divided into various types based on the clinical picture and lesion distribution. Common clinical variants of porokeratosis include disseminated superficial actinic porokeratosis (DSAP), disseminated superficial porokeratosis, linear porokeratosis, porokeratosis of Mibelli, porokeratosis palmaris et plantaris disseminata, and punctate porokeratosis; some of the less frequent subtypes of porokeratosis include eruptive bullous, follicular, genitogluteal, lichen planus-like, porokeratotic acanthoma, porokeratotic adnexal ostial nevus, and pruriginous. All forms of porokeratosis present with a centrally atrophic area that is surrounded by a well-demarcated hyperkeratotic ridge; the microscopic examination of this ridge demonstrates a cornoid lamella [1-3].

DSAP is the most common clinical variant of porokeratosis; multiple papules and plaques appear bilaterally on sun-exposed areas with a predilection for distal limbs. The lesions have a slightly reddish or brownish hue and the surrounding ridge typically appears more accentuated than the circumscribed interior. Annular or irregular configurations are characteristics as the lesions centrifugally expand [4,5].

The diagnosis of DSAP is suspected clinically; dermatoscopy and microscopic evaluation of a tissue biopsy specimen are useful aids to support and confirm the diagnosis [5-12]. An older woman with DSAP is presented. Her clinical, dermoscopic, and pathology findings are described to emphasize the features of this rare, chronic, and benign cutaneous disorder.

## Case presentation

A 67-year-old woman presented with multiple itchy red lesions on the distal arms and legs. The lesions had been present for five years. The patient complained that each year, during the summer, the itch was more intense, the redness was brighter, and the number of lesions increased. She had a history of chronic sun exposure which occurred during gardening. There was no drug history and her family history was negative for similar skin lesions.

**Figure 1:**
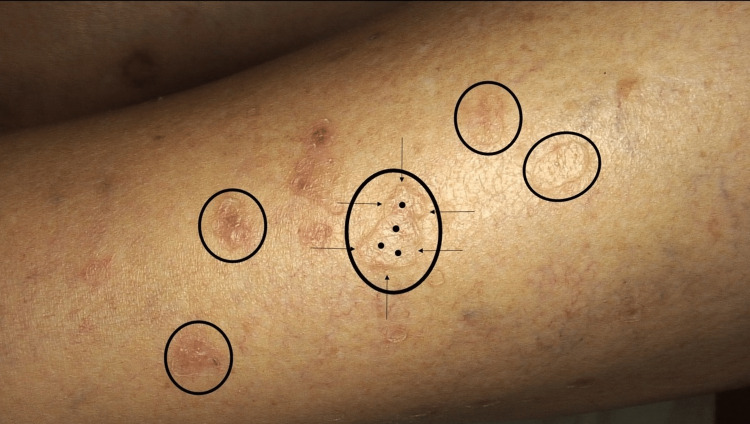
Clinical presentation of disseminated superficial actinic porokeratosis (DSAP) The left anterolateral distal leg of a 67-year-old woman has numerous annular, reddish-brown plaques and papules of DSAP (black ovals). The plaques have a hyperkeratotic border (black arrows) surrounding a central atrophic area (black dots).

A comprehensive physical examination did not reveal any systemic abnormalities. Cutaneous examination showed multiple erythematous to brownish plaques and a few papules ranging between 5 mm and 20 mm in diameter. The annular lesions were well-demarcated and had a thin, raised, keratotic margin (Figure 1).

**Figure 2:**
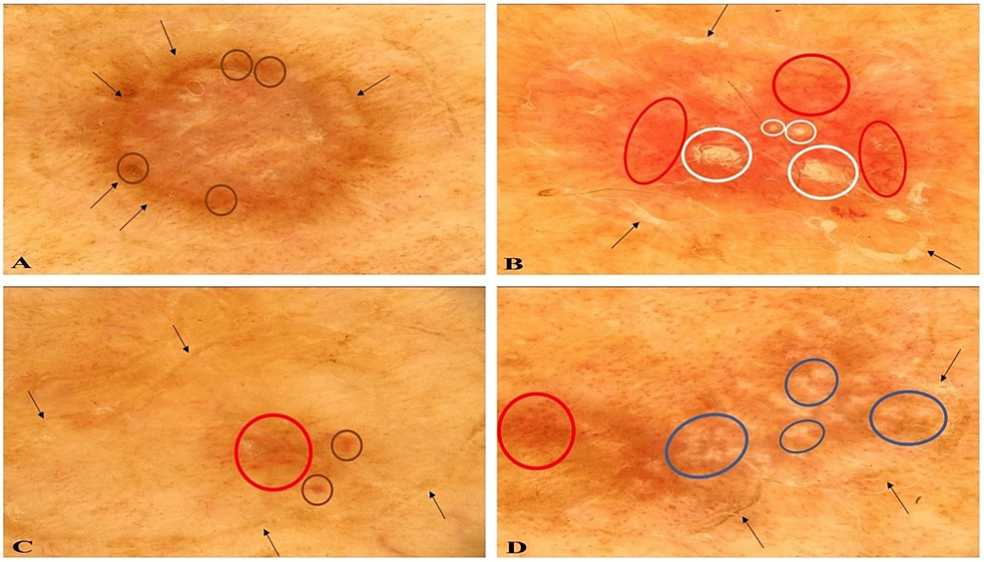
Dermatoscopic aspects of disseminated superficial actinic porokeratosis (DSAP) lesions viewed using a polarized dermatoscope A DSAP lesion shows a peripheral double-track (black arrows) with brown dots (brown ovals) (A). Another DSAP lesion shows a peripheral white and/or brown track (black arrows) with linear vessels on red background (red ovals) and white-yellow clods (white ovals); the clods represent follicular involvement of the porokeratosis lesion (B). A third lesion shows a white and brown track (black arrows) at the periphery; discreet brown dots (brown ovals) and vessels (red oval) can also be observed centrally (C). A fourth DSAP lesion shows a peripheral track (black arrows) with dotted vessels (red oval) and rosettes (blue ovals); the rosettes are an indication of sun damage in the dermis (D).

Several lesions were examined using a dermatoscope and the dermoscopy features were noted. The lesions showed either a single or double-track at the periphery; the track was white on the inner side and brown on the outer side. In addition, brown dots were densely arranged in larger or smaller areas within the peripheral white or brown lines (Figure 2A).

Other lesions had a thin, discrete border-that was either white or pale brown-with densely arranged dotted and linear vessels present centrally on a red homogeneous background. White-yellow clods were also noted in the central area. The clods represented follicular involvement of the lesion (Figure 2B).

Additional lesions showed white and brown tracks at the periphery. They also had discrete, sparse brown dots and vessels in the center (Figure 2C). Finally, some of the lesions had rosettes and dotted vessels; rosettes are frequently observed in sun-damaged skin (Figure 2D).

**Figure 3:**
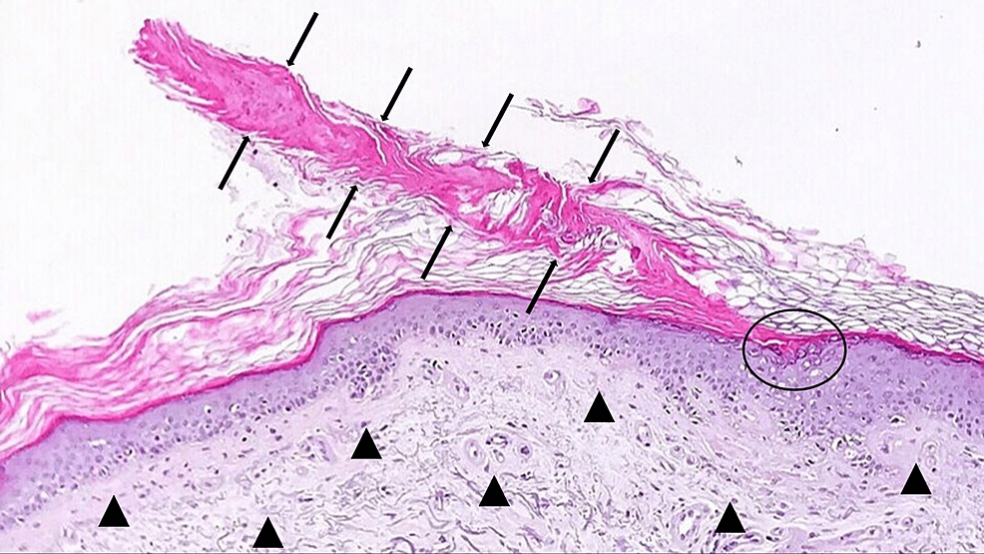
Pathologic features of disseminated superficial actinic porokeratosis (DSAP) The cornoid lamella (between black arrows) is a column of parakeratotic cells above an area of invaginated epidermis. Dyskeratotic cells can be observed in the epidermis beneath the cornoid lamella (black oval). Severe solar elastosis (black triangles) is present in the dermis (Hematoxylin and eosin, ×20).

A punch biopsy was performed from the margin of a lesion located on her left leg. Microscopic examination of the tissue specimen showed an area of hyperkeratosis that included a peripheral column of parakeratosis, the cornoid lamella, situated above part of the epidermis that contained dyskeratotic keratinocytes. The epidermis had flattening of the rete ridges and there was severe solar elastosis in the dermis (Figure 3).

Correlation of the patient’s clinical presentation, dermoscopic findings, and lesion pathology established the diagnosis of DSAP. Initial management focused on the symptomatic relief of her pruritus. She was treated with topical methylprednisolone aceponate 1 mg per g cream applied once daily for three weeks. At a one-month follow-up visit, the pruritus had resolved. However, neither the number nor the morphology of her DSAP lesions was changed.

The patient declined any other topical or systemic treatment for her condition, including cryotherapy. Photoprotective measures-such as long sleeves, trousers, and sunscreen-were initiated; it was emphasized that these measures should especially be used during the warmer months of the year when she was outside. She will also return for regularly scheduled follow-up skin examinations to evaluate for any premalignant or cancerous changes in her DSAP lesions.

## Discussion

DSAP is a benign intraepidermal condition caused by sun exposure or artificial ultraviolet radiation exposure or both in a patient who is genetically predisposed. However, other factors can also be involved in the pathogenesis of DSAP. For example, immunosuppression and aging can cause somatic homologous recombination with an ultraviolet signature in the epidermis [8,13].

Familial DSAP usually manifests during the third or fourth decade of life and exhibits an autosomal dominant mode of inheritance. The sporadic form of DSAP is characterized by a late-onset and a negative family history of the condition. In addition, congenital heterozygous mutations in genes of the mevalonate pathway have been identified as a causative factor behind both familial and sporadic cases. The patient in this report had the sporadic form; her lesions initially appeared at the age of 62 years [8,13].

DSAP lesions characteristically appear on sun-exposed skin. Facial lesions of DSAP, albeit rare, can also develop. Indeed, approximately 15% of DSAP patients have lesions on their faces [9,13].

Sun exposure is likely to be the primary etiologic factor for the development of porokeratotic lesions in the reported patient. Her lesions appeared exclusively on sun-exposed areas such as the forearms and distal legs and were always aggravated after sun exposure during the summer while gardening. The presence of rosettes, visualized using a polarized dermatoscope, within the porokeratotic lesions also supports this hypothesis; rosettes, although not DSAP lesion-specific, are a common finding in sun-damaged skin [9,13].

Porokeratotic lesions are persistent. Their evolution is marked by changes in color and shape; the changing morphology may be secondary to the degree of inflammation associated with the lesion. These changes can be observed using a dermatoscope. Indeed, investigators have described the clinical and dermatoscopic evolution of DSAP lesions from a non-atrophic lesion with a circular contour to an atrophic lesion that is polycyclic [5-7,10,12].

The initial lesions of the patient in this report appeared as round, non-atrophic brown papules; dermoscopy showed a white and brown annular double-track at the periphery and brown dots in the surrounded central area. Older, evolving lesions, had an atrophic center, and were round and red; dermoscopy showed central vessels and a peripheral white track. Finally, inactive atrophic lesions were polycyclic and skin-colored; dermoscopy showed a white track not only at the periphery but also inside the lesion.

The DSAP lesions of the reported patient also had clinical and dermoscopic features of follicular involvement. This was observed clinically as small, round keratotic areas within the porokeratotic lesions. Dermatoscopically, it presented as white-yellow clods. Follicular involvement in porokeratosis is rarely described in the literature; however, retrospective histologic studies have found this feature to be commonly encountered [11].

The histologic sine qua non of porokeratosis is the cornoid lamella. It appears as an oblique column of parakeratosis that indents the epidermis; the granular layer is either reduced or absent and there are dyskeratotic keratinocytes. The cornoid lamella corresponds clinically to the hyperkeratotic ridge. A lymphocytic inflammatory infiltrate or solar elastosis or both may be present in the dermis; the reported patient had severe solar elastosis [12,13].

Porokeratosis can be a premalignant disease. Malignant transformation occurs in 6.9% to 11.6% of patients [14]. Squamous cell carcinoma in situ and squamous cell carcinoma are the most common tumors; however, basal cell carcinoma, melanoma, and other cancers have been observed to develop in the porokeratosis lesions. Therefore, a regularly scheduled follow-up of the reported patient and her DSAP lesions is planned [14].

Disseminated porokeratosis can also be associated with other disorders. These include not only autoimmune diseases treated with systemic corticosteroids but also immunosuppressive conditions such as chronic liver disease, human immunodeficiency virus infection, and solid organ transplant recipients. In addition, hematologic dyscrasias and solid tumors have rarely been described in patients with disseminated porokeratosis [15].

There are several treatment options that have been described for patients with DSAP. They include cryotherapy, diclofenac, 5-fluorouracil, imiquimod, photodynamic therapy, retinoids, and vitamin D3 analogs. Laser therapy-including carbon dioxide laser and fractional photothermolysis-has also been performed. Even superficial radiotherapy-using Grenz ray-has been used [6,16-18].

Topical corticosteroids for individuals with pruritic lesions, similar to the patient in this report, have also been used [16-18]. In addition, also similar to the patient in this report, some individuals elect to only observe their lesions. However, since there is a potential risk of malignancy appearing in the DSAP lesions, regularly scheduled follow-up clinical evaluation of the lesions should be performed [14].

## Conclusions

DSAP is a keratinizing disorder that typically occurs on sun-exposed areas. The woman described in this report had late-onset sporadic DSAP. Her diagnosis was suspected clinically and confirmed by microscopic examination of a tissue biopsy specimen. Dermatoscopic evaluation supported the diagnosis of the patient’s DSAP. Indeed, dermatoscopy is a useful and non-invasive diagnostic procedure that can be used when the diagnosis of porokeratosis is being considered to exclude other conditions. There is a risk of malignancy developing in DSAP lesions. Photoprotective measures should be encouraged in order to minimize the risk of further skin damage. Also, patients should return on a regular basis for monitoring of their lesions.
